# When Knowledge Is Not Enough: Applying a Behavioral Design Approach to Improve Fever Case Management in Nigeria

**DOI:** 10.9745/GHSP-D-22-00211

**Published:** 2022-12-21

**Authors:** Faraz Haqqi, Angela Acosta, Sriram Sridharan, Emily Zimmerman, Temitope Ogunbi, Eno’bong Idiong, Uwem Inyang, Foyeke Oyedokun-Adebagbo, Jose Tchofa, Nene Diallo, Emma Mtiro, Chukwu Okoronkwo, Bolatito Aiyenigba

**Affiliations:** a Breakthrough ACTION, ideas42, New York, NY, USA.; b Breakthrough ACTION, Johns Hopkins Center for Communication Programs, Baltimore, MD, USA.; c Breakthrough ACTION, Johns Hopkins Center for Communication Programs, Abuja, Nigeria.; d U.S. Agency for International Development, Abuja, Nigeria.; e U.S. President’s Malaria Initiative, Abuja, Nigeria.; f Nigeria National Malaria Elimination Programme, Abuja, Nigeria.

## Abstract

**Background:**

We sought to encourage health care providers to adhere to national malaria case management guidelines. This requires them to conduct malaria parasite tests for every patient presenting with a fever and provide malaria treatment only to those who test positive for malaria. Our goal was to make it easier for providers to follow guidelines by addressing drivers of nonadherence uncovered through facility observations and interviews with staff and clients.

**Implementation and Monitoring:**

The case management interventions were piloted in 12 public health facilities in Akwa Ibom, Kebbi, and Nasarawa states in Nigeria between October and December 2019. Participating facilities included 1 hospital and 3 primary health centers in each state. Relevant changes included the following: (1) providers at each facility participated in facilitated discussions to correct misconceptions about the reliability of malaria test kits; (2) testing procedures were integrated into existing triage systems; (3) treatment algorithms were integrated into medical record forms; (4) providers were issued pictorial brochures outlining danger signs to share with clients, together with instructions for when to seek further care; and (5) a process was created for facilities to monitor their own adherence to guidelines.

**Lessons Learned:**

The lessons learned include: (1) disentangling the drivers of behavior allows for more targeted solutions, (2) solutions that streamline processes for overburdened providers allow them to redirect their attention and efforts where they can be most impactful, and (3) changing staff perceptions of workplace norms can support a holistic and sustained approach to behavior change.

## Abstract (precis)

Analyzing fever case management through a behavioral lens can lead programs to solutions that differ from conventional approaches in terms of type and deployment method.

## Teaser

**Key Findings:**

- Health care providers encounter various behavioral barriers when trying to follow case management guidelines—even those providers who are aware of proper case management protocols and intend to follow them.
- Understanding that behavioral drivers varied between individuals allowed for the design of a multipronged intervention to address the combination of factors influencing provider behavior.
- The intervention streamlined processes for overburdened providers, allowing them to focus their attention and efforts where they are most impactful.
- In addition to correcting provider misconceptions, the solutions also created a shared understanding of the reliability of malaria rapid diagnostic tests among providers and reset expectations with respect to patient case management and facility-wide adherence to national guidelines.

**Key Implication:**

- In challenging environments, a behavioral design approach can help improve case management practices by focusing solutions on removing barriers to create an environment more conducive to all of the tasks that case management requires, equipping providers with tools to better navigate the barriers they encounter, and establishing workplace norms to support and sustain changes in provider behavior.

## BACKGROUND

Appropriate diagnosis and treatment of fever are essential to the reduction of morbidity and mortality and to the appropriate use of medicines. In Nigeria, malaria is likely to be overdiagnosed and overtreated by health care providers who may also be ill-equipped to diagnose other illnesses.^1^^,^^2^ The Breakthrough ACTION project team sought to complement existing efforts to curb malaria by taking a behavioral design approach^3^—developing new solutions for the specific contextual and cognitive factors driving provider behavior—to improve fever case management in Nigeria.

### Problem and Significance

Our goal was to encourage health care providers to: (1) conduct malaria parasitological tests for every patient presenting with fever or a history of fever, (2) provide malaria treatment only to those who test positive for malaria—usually with artemisinin-based combination therapy (ACT), and (3) assess nonmalaria clients for other potential causes of fever. Although the National Guidelines for Diagnosis and Treatment of Malaria 2015^4^ emphasize the need for parasitological confirmation before prescribing antimalarial drugs, many health care providers (23%–51%) in the public sector prescribe ACTs to clients who test negative for malaria.^5^^,^^6^ The 2018 Nigeria Demographic and Health Survey also found that of 7,466 children younger than 5 years with fever in the 2 weeks preceding the survey, only 1,030 had blood drawn for malaria testing, yet 3,244 children took antimalarials (and over half of those children took ACTs).^7^ This suggests that testing and treating based on test results is not widespread.

There are important health consequences to deviating from these guidelines. Providers who assume clients have malaria may overlook illnesses with similar symptoms, like pneumonia, that are among the leading causes of child mortality in Nigeria.^8^ Prescribing and dispensing antimalarials to clients without malaria can also result in fewer medicines being available to patients who need them and would benefit from their use.

## BEHAVIORAL DESIGN APPROACH

The project team adopted a behavioral design approach to gain new insights into the contextual and cognitive factors driving provider nonadherence to the national testing and treatment guidelines and to design solutions targeting those factors.

The project team adopted a behavioral design approach to gain insights into factors driving provider nonadherence to the national guidelines and to design solutions targeting those factors.

To understand why providers might not test clients with fever or with a history of fever for malaria and why they might disregard test results when making treatment decisions, beginning in September 2018, we interviewed 92 health facility staff and 56 clients at 29 hospitals and clinics in Akwa Ibom, Kebbi, and Nasarawa states in Nigeria. Staff were asked to describe their approach to fever case management and decision making, while clients were asked to describe their approach to care-seeking and their experiences at facilities. We also conducted observations of client flow and clinical consultations in facilities to understand how a provider’s testing and treatment choices might be influenced by their environment. Findings suggested that even well-informed and well-intentioned providers can fail to adhere to the guidelines due to established cognitive principles, including (1) limited attention, (2) tunneling, (3) base-rate neglect, and (4) salience. For example, factors like high client loads discourage providers from requesting time-consuming malaria tests for every fever case (limited attention) in an effort to see as many clients as possible (tunneling). The knowledge that malaria is prevalent leads providers to assume that some malaria rapid diagnostic test (mRDT) results are false negatives, overlooking the fact that mRDTs have high sensitivities and specificities and therefore have low false-negative rates (base-rate neglect). Providers may also face pressure from clients to prescribe treatments. Lacking any other sources of feedback or accountability (salience) for their treatment decisions, providers may find it challenging to base treatments strictly on clients’ test results.^9^

To ensure that solutions to address these behavioral drivers of nonadherence would be appropriate, stakeholder input was incorporated into every stage of their development. Nineteen stakeholder institutions, including nongovernment partners and government representatives from the national, state, and local levels, contributed to solution development through a 3-day codesign workshop. The intention of the solutions was to make it easier—psychologically and practically—for providers to follow guidelines. Workshop participants generated more than 300 initial ideas that were distilled into a package of complementary designs, each of which responded to distinct needs in the fever case management process. Prototypes of each design were prepared with further inputs from stakeholders, after which clients and staff at 12 health care facilities interacted with the prototypes and contributed feedback. Their feedback was incorporated into the designs through an iterative revision process.

The final designs prioritized approaches requiring fewer resources and limited technical capacity, which would be easier to deploy at scale (Figure). First, providers at each facility participated in a facilitated discussion to correct misperceptions about the reliability of mRDTs (Figure, Step A). Providers were also offered regular feedback on the frequency with which they stray from guidelines (Figure, Step B) to help them recognize the magnitude of the problem and the role of their behavior in causing it. Teams comprising project staff and government personnel conducted monthly supportive supervision visits, during which they also surveyed facility records and compared the number of positive malaria test results to the number of ACTs issued. These numbers were recorded and updated monthly on a prominently placed poster within the facility. They also provided reliable data for monitoring purposes.

**FIGURE.**
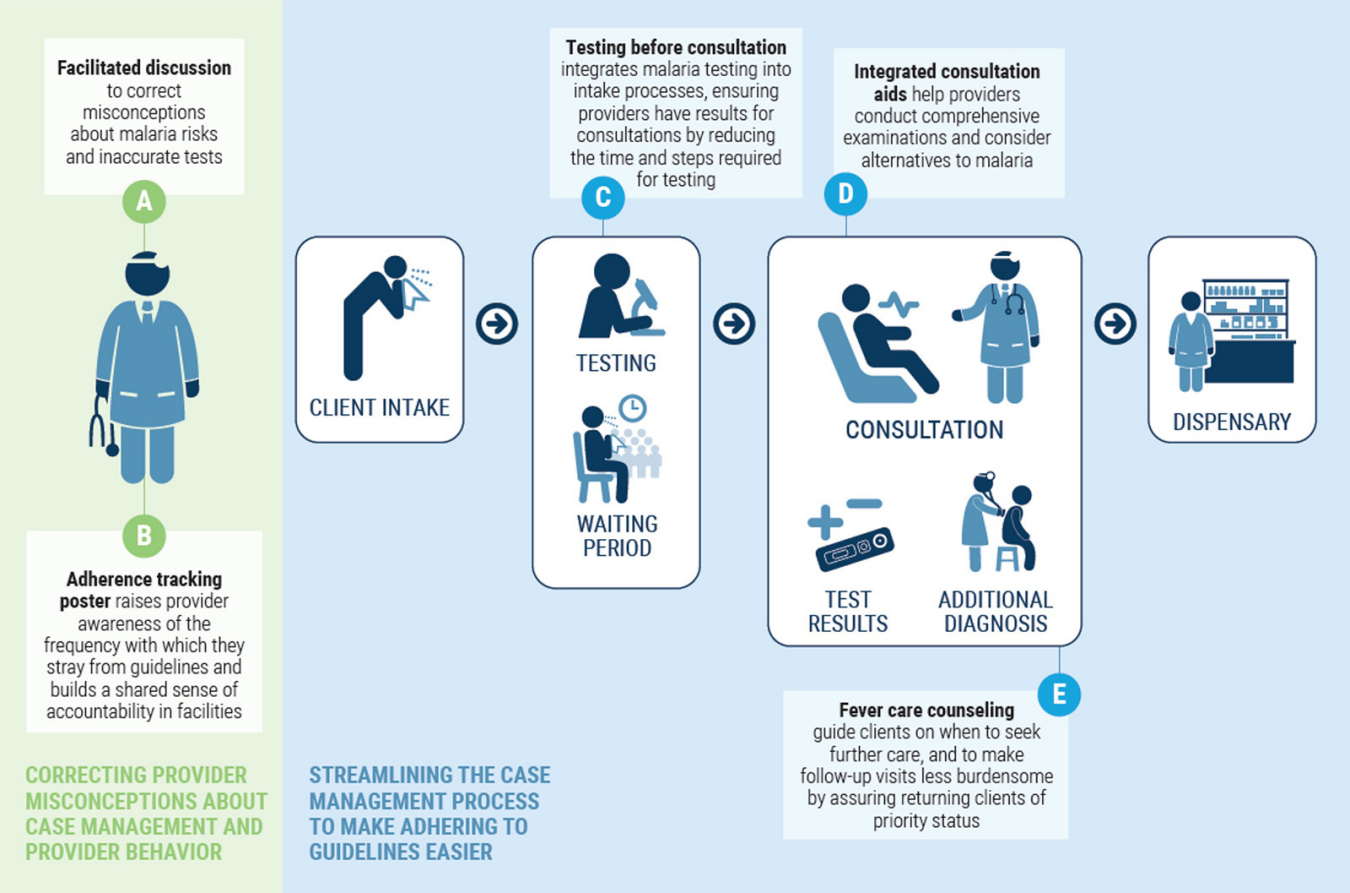
Building Behavioral Design Interventions Into the Fever Case Management Process

Next, testing procedures were integrated into intake/triage systems before clients consulted with providers (Figure, Step C). This was done to reduce the additional time and steps required for testing and to ensure that providers could review test results when they first met with clients and use those results to form an initial diagnosis. Testing before provider consultations has previously proven effective at regulating presumptive treatment in Ghana.^10^ Consultation aids were also integrated into client intake forms (Figure, Step D) to make it easier for providers to conduct comprehensive evaluations of pediatric clients according to Integrated Management of Childhood Illness guidelines and to encourage them to consider common illnesses besides malaria. Finally, to alleviate provider concerns about turning away clients without clear diagnoses, they were given counseling tools to share with clients, along with instructions for when to seek further care (Figure, Step E).

To ease implementation, the project team created a facilitation guide for the initial discussion with providers; checklists for comprehensive supportive supervision visits that also guided supervisors to calculate the metrics to be shared as feedback; and a poster template that facilities could use to share feedback with their staff. The project team also created a 1-page checklist to aid consultations and a short counseling script that could be distributed to clients as handouts.

### Ethical Approval

The formative research to identify behavioral barriers and the piloting of solutions were both approved by Institutional Review Boards at the Johns Hopkins Bloomberg School of Public Health and by local boards in Akwa Ibom, Kebbi, and Nasarawa states in Nigeria.

## IMPLEMENTATION AND MONITORING

The solutions were piloted in 12 health facilities over 3 months, from October to December 2019, including in hospitals and primary health centers in each of the 3 states. To maintain a focus on provider behavior, the solutions were piloted at facilities that were unlikely to face additional challenges in acquiring the resources and supplies they need for malaria case management due to support they received from international donors. The selected facilities all met the criteria of (1) being accessible to supervisors, (2) experiencing a medium to high client volume, (3) having a history of poor adherence to testing and treatment guidelines, and (4) having sufficient staffing in the local government area for supervision. Facilities’ adherence to guidelines was calculated as the ratio of ACTs prescribed to the number of positive malaria test results recorded at a facility. Facility adherence less than 100% indicated a facility issued more ACTs than warranted by the number of positive malaria test results recorded at that facility. Facility adherence greater than 100% indicated a facility issued fewer ACTs than required to treat positive malaria test cases.

Facilities in every state converged toward 100% adherence over the 3-month pilot period, as was desired (Table 1). On average, primary health centers demonstrated higher levels of adherence than hospitals. However, hospitals demonstrated greater improvements in adherence over the course of the pilot than did primary health centers, suggesting that larger facilities may have more potential to benefit from interventions that streamline processes and establish new workplace norms.

**TABLE 1.** Monitoring Data for Behavioral Design Interventions to Improve Fever Case Management in Nigeria

	**Month 1**		**Month 2**		**Month 3**	
	**Facilities, No.**	**Facility Adherence,**^a^ **%**	**Facilities, No.**	**Facility Adherence,**^a^ **%**	**Facilities, No.**	**Facility Adherence,**^a^ **%**
Primary care centers	9	92	9	104	9	99
Hospitals	2	42	3	92	3	122

a Average facility adherence was calculated as the ratio of artemisinin-based combination therapy regimens prescribed to the positive malaria test results.

Hospitals demonstrated greater improvements in adherence over the course of the pilot than did primary health centers.

Provider responses to a knowledge and attitude test designed to measure common misperceptions about malaria diagnosis also demonstrated improvements in knowledge and trust in test kits after the pilot compared to tests before the pilot (Table 2). Although these trends do not offer conclusive evidence of impact, the improvement in knowledge, attitudes, and behavior suggests that the solutions may have had their intended effect in improving case management practices. We did not conduct tests for statistical significance due to the small sample of facilities.

**TABLE 2.** Provider Knowledge and Attitudes Test Results Before and After Behavioral Design Interventions to Improve Fever Case Management in Nigeria

	**Correct or Desirable Responses to Knowledge Questions**	
	**Before Pilot, No. (%)** **(N=207)**	**After Pilot, No. (%)** **(N=127)**
Poor (hot) storage conditions can cause RDTs to give false negative results (false)	24 (12)	29 (23)
Malaria RDTs can miss malaria in febrile children if the child has low parasite levels (false)	76 (37)	72 (57)
Number of children with malaria has decreased in the past 10 years (true)	98 (48)	94 (74)
Any laboratory scientist who knows how to use a microscope can diagnose malaria (false)	33 (16)	32 (25)
Your colleagues believe that positive RDT results can be trusted (yes)	177 (86)	121 (95)
Your colleagues believe that negative RDT results can be trusted (yes)	124 (61)	97 (76)

Abbreviation: RDT, rapid diagnostic test.

## LESSONS LEARNED

We learned the following lessons from pilot testing the fever case management interventions.

Disentangling the drivers of behavior allows for more precisely targeted solutions. Interviews with providers and observation of facilities revealed that there is no single reason why providers sometimes failed to adhere to guidelines. Drivers of nonadherence varied with individual characteristics of providers—such as cadre, number of years of experience, and perceptions of malaria and mRDTs—as well as the size, staffing, and operating hours of facilities. Understanding these variations allowed us to design a multipronged intervention to address a combination of factors influencing provider behavior across facilities, with a greater likelihood of effecting behavior change across individuals.

Streamlining processes for overburdened providers can allow them to redirect their attention and efforts where they can be most impactful. Research suggests most people intuitively formulate solutions that require introducing new elements into a context, systematically overlooking subtractive changes focused on removing problematic elements from a context.^11^ Yet, solutions that remove obstacles—eliminating hassles or simplifying complex procedures—can be just as powerful, allowing providers to be more effective and productive in their work. By streamlining malaria testing, giving providers clients’ malaria test results at the start of their consultation, and integrating algorithms for nonmalaria fever cases into providers’ case notes, we intended to reduce the cognitive burden on providers of having to await confirmation of diagnosis (in suspected malaria cases). This, in turn, would allow providers to devote more of their attention to diagnosing other possible illnesses, especially in cases that were confirmed not to be malaria. It should be noted that solutions that themselves require effort to administer may prove to be less impactful, especially when evaluated over a longer period than the 3 months for this project.

Changing staff perceptions of workplace norms can support a holistic approach to behavior change. In initial codesign activities, some stakeholders proposed familiar, information-based approaches that leveraged conventional tools like standard operating procedures and training to remind health care providers of malaria case management guidelines and to convince providers of the importance of adhering to those guidelines. However, the feedback and perspectives shared by providers also suggested that providing information alone was unlikely to change behavior, as many of those who failed to follow guidelines already understood the requirement to test for malaria before treating clients. In addition to correcting provider misconceptions, the solutions also sought to change norms within the workplace, by bringing providers together to create a shared understanding about reliability of mRDTs and by promoting a shared sense of accountability for facility-wide adherence by highlighting discrepancies between providers’ values and behaviors.

## CONCLUSION

The behavioral design approach helped uncover persistent barriers to case management affecting the behavior of even those providers who were aware of proper case management protocols and intended to follow them. By designing a package of complementary, mutually reinforcing solutions to address those specific barriers—including limited time and attention for requesting malaria tests for every fever case, tunneling on malaria’s prevalence, and misperceptions about the reliability of test results stemming in part from base-rate neglect of low mRDT false-negative rates, as well as pressure from clients and a lack of alternate sources of feedback or accountability—we sought to create an environment in which providers found it faster, easier, and more comfortable to follow fever case management guidelines.

The project’s findings suggest the potential for behavioral solutions to improve case management practices without the need for substantial additional inputs. Behaviorally informed solutions can help providers fulfill the challenging roles with which they have been tasked by removing barriers to create an environment more conducive to performing all the tasks that case management requires (and which providers are by and large already motivated to do); equipping providers to better navigate their environment and focus their time and attention where they are most needed; and encouraging workplace norms to support and sustain changes in behavior. Further research, including a trial to measure longer-term impacts and an evaluation of the solutions individually or in different combinations, would also yield useful insights about the potential of these specific solutions.

## Notes

**Peer Reviewed**

**First published online:** December 15, 2022.
